# Infraclavicular arterio-arterial prosthetic loop is a safe and effective vascular access technique for haemodialysis in frail patients: a prospective observational study

**DOI:** 10.11604/pamj.2021.40.245.29390

**Published:** 2021-12-21

**Authors:** Ahmed Mohammed Ahmed Abdel Rahim, Alexander Bush, Aml Ahmed Sayed Ahmed, Aml Mohammed Soliman, Mohammed Ahmed Mohammed Ismail

**Affiliations:** 1 Department of Vascular Surgery, Aswan University Hospital, Aswan, Egypt,; 2 Department of Vascular Surgery, Derriford Hospital, Plymouth, United Kingdom,; 3 Department of Nephrology, Aswan University Hospital, Aswan, Egypt,; 4 Department of Cardiology, Aswan University Hospital, Aswan, Egypt

**Keywords:** Arterio-arterial prosthetic loop, vascular access, central venous occlusion, exhausted vascular access, peritoneal dialysis

## Abstract

Vascular access failure causes significant morbidity among end stage renal failure patients. With the increased life expectancy and frailty of those patients, maintaining vascular access became a great challenge. In this study, we assess the short and midterm outcomes of infraclavicular arterio-arterial prosthetic loop (IAAPL) as vascular access for haemodialysis in frail patients who have exhausted conventional vascular access methods. A prospective observational study of 43 patients undergoing IAAPL was conducted in a single centre between May 2017 and March 2020. Primary, assisted primary and secondary patency rates were recorded in addition to complications and patient compliance with access. The achieved primary, assisted primary and secondary patency rates at 6 months are 87.5%, 95%, 97.5% respectively, at one year, corresponding rates were 75%, 83.3%, 94.4% and at 18 months they were 68.6%, 77.1%, 85.7% respectively. There was no procedure related mortality and life-threatening complications during the study period. So we can assume that infraclavicular AAPL is a safe and effective method of obtaining alternative vascular access for hemodialysis in frail patients for whom the conventional vascular access for hemodialysis is not suitable or contraindicated.

## Introduction

End-stage renal disease (ESRD) is a huge public health problem which is associated with significant morbidity, mortality, and cost [1]. Early diagnosis and nephrology involvement in the management of Chronic Kidney Disease (CKD) has significantly improved outcomes. As a result, older patients with increasing frailty represent a large proportion of ESRD patients [2,3]. Frailty can be broadly defined as a composite condition which includes low physical activity, exhaustion, weight loss, repeated hospital admissions, increased risk of falls and cognitive impairment and is associated with multiple morbidities [4,5]. The main types of vascular access are autogenous arterio-venous fistula (AVF), arterio-venous graft (AVG), double lumen catheter and arterio-arterial access [3]. Alternatives to these include right atrial bypass grafting or axillo-renal arterio-venous bypass grafting but these are complex, morbid procedures and are not suitable for frail patients [6]. There is a group of frail patients with ESRD who have exhausted traditional access options or who cannot tolerate the additional cardiac loads associated with high-flow arterio-venous (AV) access. There is no consensus on what access type is optimal for these patients [7,8]. Arterio-arterial access represents one promising option for frail patients. This access method was first described in 1976 where two cannulas were connected to the radial artery for dialysis access [9]. Further arterio-arterial access methods have been described since at various anatomical sites; brachial artery, superficial femoral artery, axillary artery and (bovine) carotid artery [10-17]. A 2018 systematic review showed good primary and secondary patency rates [18]. This study aims to evaluate AAPL as access for haemodialysis in frail patients. We consider quality of life factors along-side the traditional measures of patency, morbidity and mortality.

## Methods

**Study design and setting:** a prospective observational study was conducted between May 2017 and March 2020 including 43 patients with ESRD needing vascular access for haemodialysis at Aswan University Hospital. Patients underwent infraclavicular arterio-arterial prosthetic loop (IAAPL).

**Study population:** all patients were initially assessed with detailed history and physical examination, arterial and venous duplex, echocardiography computed tomographic arteriography (CTA) and computed tomographic venography (CTV). Frailty was assessed using the rockwood clinical frailty score. Inclusion criteria were one or more of the following: heart failure refractory to therapy (ejection fraction ≤40%), cardiological assessment indicating that AV access would not be tollerated, exhausted upper limb options for conventional arteriovenous fistula, deep veins (subclavian, internal jugular, and femoral veins) with high grade stenosis or occlusion (<70% in diameter, >4cm long), untreatable venous outflow obstruction, peripheral vascular disease with inadequate limb perfusion (risk of Steel syndrome). Exclusion criteria were: concurrent widespread infection of the upper limb, stenosis in arterial inflow or outflow, current or past upper limb ischemia.

**Figure 1:**
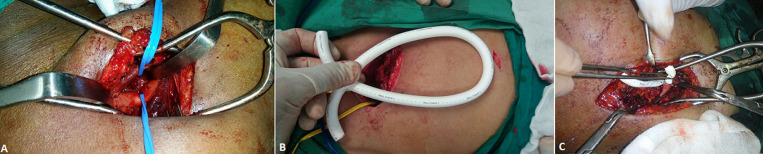
intra-operative images demonstrating; A) infraclavicular exposure of the axillary artery; B) chest wall loop graft; C) end-to-end anastomosis of the graft

**Procedure:** all procedures were performed under general anaesthesia. Two grams of 3^rd^ generation cephalosporin antibiotic were administered with induction of anesthesia. A 10cm infraclavicular transverse incision was made and subcutaneous tissues were dissected. Pectoralis minor muscle was divided near its insertion. The first part of axillary artery was exposed. The artery was transected and a Polytetrafluoroethylen (PTFE) graft (Le maître) with a 30-40cm length a 6-8mm diameter (matched to the diameter of the native artery) was interpositioned (end-to-end) using a 6/0 polypropylene suture (Ethicon) after configuration of a subcutaneously tunnelled loop on the chest wall (Figure 1). The mean operation time was 102 minutes. Low molecular weight heparin at therapeutic dose was administered once a day for two days then was replaced by lifelong dual antiplatelet therapy (aspirin 75mg and clopidogrel 75mg). Patients were discharged after 3 days unless they suffered complications. The first needle puncture of the graft was not carried out until at least two weeks after the procedure. The graft we assessed during dialysis sessions to ensure its patency. Written instructions were sent to the nephrologists to inform access use. Temperature of the reinfused blood must be adjusted to avoid arterial vasospasm, pump power (250-280ml/min), pressure over the puncture site (15 minutes after the removal of the needle), cautions about infusion of medications (intra-arterial injection), and the heparin administration until 30 minutes before the end of haemodialysis were specified. In addition, instructions about puncture sites change (stepladder technique) were given.

**Data collection:** all patients were followed up in the vascular unit at 6, 12, and 18 month intervals or if there was any urgent clinical indication. Surveillance including graft patency, clinical examination and duplex ultrasound scanning were routinely conducted. If there was any sign of graft dysfunction, duplex ultrasound was performed as the first-line investigation. Interventions to retain patency were performed according to any problems detected. Data was collected prospectively including demographics, comorbidities, rockwood frailty score, indications for AAPL, patency rates, dialysis schedules, compliance, morbidity and mortality.

**Definitions:** study outcome measures were defined according to the Society for Vascular Surgery (SVS) reporting standards and the European Society for Vascular Surgery (ESVS) clinical practice guidelines, namely: a) primary patency defined as the interval from the time of access placement until any intervention designed to maintain or to restore patency, access abandonment, or the time of measurement of patency; b) assisted primary patency defined as the interval from the time of access placement until access abandonment or the time of measurement of patency including intervening manipulations (surgical or endovascular) designed to maintain patency; and c) secondary patency defined as the interval from the time of access placement until access abandonment or the time of measurement of patency including intervening manipulations (surgical or endovascular) designed to restore patency [19]. Secondary outcome measures were morbidity and mortality and compliance with dialysis.

**Statistical analysis:** the collected data was revised, coded, tabulated and introduced to a personal computer (PC) using Statistical package for Social Science (SPSS 25). Analysis was performed according to the type of data obtained for each parameter. Mean, standard deviation (± SD) and Range were presented for parametric numerical data, while median and interquartile range (IQR) for non-parametric numerical data. Frequency and percentage were presented for non-numerical data. Patency rates were analysed on an intention-to-treat basis using Kaplan-Meier survival curve.

**Ethical consideration:** ethical approval for the study was given by the Professional Ethics and Scientific Research Committee at Aswan University Faculty of Medicine and written informed consent was taken from all patients prior to the procedure.

## Results

**General characteristics of the study population:** five hundred and forty-two (542) patients were assessed for vascular access for haemodialysis. Of these patients 43 met inclusion criteria and were enrolled in the study. No patients met exclusion criteria. Baseline characteristics are presented in Table 1. Patients had a median age of 65 years (IQR 57-70, range 50-80). Their rockwood clinical frailty score for the ranged from 6-8 with median of 6. The main indications for IAAPL were absence of accessible superficial veins (74.4%), heart failure (NYHA class III) (46.5%), bilateral central vein occlusion (46.5%), contraindication for conventional AVF or AVG (32.6%) and history of steel syndrome after AVF (18.6%). 90.7% of patients had more than one indication. 27 (62.8%) of the patients underwent right sided operations.

**Table 1:** baseline characteristics

	N /mean	% / SD	Median (IQR)	Range
Age	-	-	65 (57- 70)	50 - 80
Male/female	21/22	48.8%/51.2%		
Diabetic	26	60.5%		
Comodities				
Hypertension	23	53.5%		
IHD/HF	16	37.2%		
Ejection fraction	-	-	33% (25% - 37%)	(15% - 50%)
Dyslipidaemia	24	55.8%		
Smoking	20	46.5%		
CVE	13	30.2%		
Liver disease	16	37.2%		
PVD (LL)	15	34.9%		
Rookwood clinical frailty score	-	-	6 (6 - 7)	(5 - 8)

**Figure 2:**
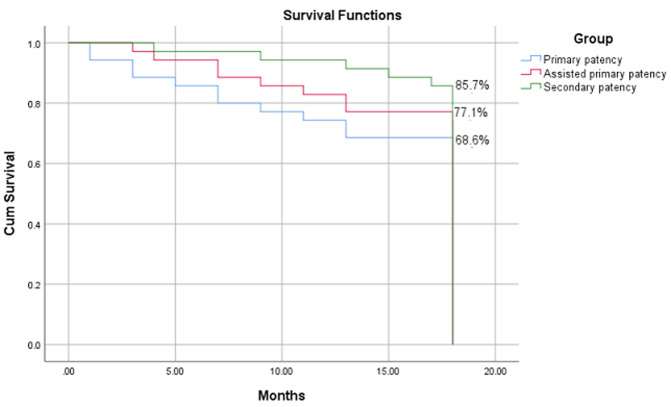
curves calculated using the Kaplan-Meier method for primary and secondary patency and their negative standard error bars

**Patency:** the achieved primary, assisted primary and secondary patency rates are demonstrated using the Kaplan-Meier curve (Figure 2). At 6 months the rates were 87.5%, 95%, 97.5% respectively, at one-year, corresponding rates were 75%, 83.3%, 94.4% and at 18 months they were 68.6%, 77.1%, 85.7%.

**Compliance:** the first cannulation day ranged between 10 and 25 days with a mean of 14.49 ± 4.1. Pre-operative dialysis history for the patients is summarised in Table 2. Eighty-three point seven percent (83.7%) of the patients had an increase in their dialysis schedule time by a mean of 2.06±1.14 hours weekly. Compliance was generally good (83.72%). In patients with poor compliance the reasons cited were need for long compression time (n=4) and difficulty of cannulation due to obesity or pain (n=3).

**Table 2:** dialysis schedules

		N / mean	% / SD	Median (IQR)	Range
**Pre-operative dialysis schedules**					
Prior Access procedures		3.72	1.93	4 (2 - 5)	(0 - 8)
Prior central vein cannulation		6.02	4.09	5 (3 - 8)	(0 - 25)
Dialysis schedule prior to surgery (per week)	2 times 3 hrs each	1	2.3%		
	2 times 3.5 hrs each	1	2.3%		
	3 times 3 hrs each	26	60.5%		
	3 times 3.5 hrs each	15	34.9%		
**Post-Operative Changes to dialysis schedule**					
Dialysis time difference	The same	7	16.3%		
	Increased	36	83.7%		
Dialysis time difference (by hours)	2.06	1.14	3 (1.5 - 3)	(0 - 3)	

**Morbidity and mortality:** morbidity data is summarized in Table 3. Six patients (14%) developed thrombosis within their grafts at mean time 8.67±3.88 months and underwent a total of 7 thrombectomy procedures (5 successful, 2 failed). All cases were identified incidentally during dialysis sessions and no patients had ischaemic manifestations. Four patients developed graft infections (9.3%). One patient responded to antibiotic therapy and three patients required eventual graft explantation. Arterial reconstruction was not possible in two cases due to severity of infection and tissue friability but no peripheral tissue ischaemic manifestations were observed during the study period. Four of the patients (9.3%) developed hematomas at a mean time of 2.26±2.21 months. All of them were successfully treated surgically and their grafts were preserved. Incorrect decannulation and compression techniques are thought to be the cause due to correlation between the cases and specific nursing staff. One patient suffered ischaemic monomelic neuropathy which was managed medically in collaboration with the neurology team and access preserved. No aneurysmal dilations were encountered in the study and there were no distal embolization or ruptures. Eight of the patients (18.6%) died during the study from causes not related to the access at mean of 7.38±3.78 months, with overall survival 81.4%.

**Table 3:** morbidity

	N / mean	% / SD	Median (IQR)	Range
Infection	4	9.3%		
Infection (by month)	7.75	7.63	6.5 (1.5 - 14)	(1 - 17)
Peripheral limb ischaemia	0	0%		
Aneurysmal dilatation	0	0%		
Haematoma	4	9.3%		
Haematoma (by month)	2.26	2.21	2 (0.52 - 4)	(0.03 - 5)
Rupture	0	0%		
Ischaemic monomelic neuropathy	1	2.3%		
Heart failure exacerbation	0	100.0%		
Thrombosis	6	14.0%		
Thrombosis (by month)	8.67	3.88	8 (7 - 13)	(3 - 13)

## Discussion

The number of patients with end-stage renal disease (ESRD) requiring haemodialysis is rising worldwide as is their prognosis and life expectancy. In the context of ESRD maintenance of vascular access represents access to life [1]. Arterio-venous fistulaand AVG represent the first- and second-line access for haemodialysis however an increasing number of patients have exhausted these options or have contra-indications due to increasing frailty. Arterio-arterial prosthetic loop or straight graft represents one possible access option for these patients, but studies of these techniques are relatively few and patient numbers are relatively low. This case series improves our understanding of clinical and quality of life outcomes of this technique. Good patency rates and compliance with therapy were achieved. For the primary outcome measures of primary, assisted primary and secondary patency rates, our results were consistent with other published series for all types of arterio-arterial access methods (Table 4) [20-23]. For comparison AV fistula primary, assisted primary and secondary patency rates at 1 year were found to be 64%, 73% and 79% respectively in a recent meta-analysis [24]. Arterio-arterial prosthetic loop is still considered a tertiary vascular access method and results should therefore be compared with central venous catheters (CVCs). Multiple studies assessing CVCs as permanent access for dialysis show higher incidence of complications and worse patency rates and quality of life [25-27]. When designing the study, consideration was given to the choice of anatomical site for the AAPL. Brachial and femoral sites have also been studied. We chose the axillary artery due to its wide diameter and high flow rate compared with the brachial artery, decreasing IAAPL thrombosis risk. Additionally, previous fistula creation within the arm limits the amount of healthy skin that is available to cover a brachial AAPL.

**Table 4:** comparison of results with other studies of AAPL

Study	N=	6 Months			12 Months			18months		
		Primary	Assisted primary	Secondary	Primary	Assisted primary	Secondary	Primary	Assisted primary	Secondary
Present study	43	87.5%	95%	97.5%	75%	83.3%	94.4%	68.6%	77.1%	85.7%
Allam	45	100%	-	100%*	93%	-	66.6%*	77.5%	-	-
Fareed	15	-	-	-	73.3%	-	86.6%	-	-	-
Zanow	31	-	-	-	73%	-	96%	-	-	-
Ali	89	87.5%	90.9%	97.7%	71.5%	79.5%	93.2%	-	-	-
Khafagy	35				87.9%		90.7%	-	-	-

The wide, flat shape of the anterior chest wall allows the loop to sit in an orientation which reduces the risk of graft kinking or stenosis. The main disadvantage to IAAPL is that surgery required general anaesthesia which does carry risk in frail patients. Although theoretically feasible, it was not felt that patients would tolerate the intervention well under local anaesthesia and it was therefore not attempted. Quality of life has rarely been the focus of access studies, which instead tend to use morbidity and mortality as the primary outcome measures. It is becoming increasingly evident however that patients place higher priority on quality of life than on traditional clinical outcomes [19]. In the present study, patient compliance, or comfort during dialysis was investigated. In 69.8% of the patients were compliant with the access with 7 patients reporting discomfort, either due to difficult cannulation or due to long compression time. In previous studies, dialysis blood flow rates of >400 mL/min were associated with painful reperfusion. This could be caused by the effect of the high pressures on the arterial wall. In studies where a dialysis flow rate of 300mL/min was used painful reperfusion was not observed [28]. In this study therefore the desired extracorporeal dialysis flow rate was set at 260-280mL/min and painful reperfusion was observed above a rate of 300mL/min. Although dialysis time increased by 2.06 ± 1.14 hours on average to compensate for the decrease in the pump flow rate, it did not affect patient compliance.

In this study, observed rates of the most common morbidity associated with these fistulas (thrombosis, infection and hematoma/pseudo aneurysm) were slightly lower than those seen in similar studies (14%, 9.3% and 9.3% compared with 24.4-46.7%, 6.7-20% and 13.3-15.6% respectively) although the interpretation of this is limited by the small numbers of patients in each study and differing lengths of follow-up. Studies with longer follow-up periods (up to 4 years) reported a greater number of post-operative complications. Post-operative survival in comparable series ranged from 71% to 93 % [29] compared with 81.4% in this study. Importantly, we found that patients tolerated graft thrombosis and explantation well and did not develop signs of limb threatening ischemia requiring urgent intervention. This is due to good collateralization of the upper limb. This is one drawback to femoral AAPL where studies have shown higher rates of limb ischaemia [29]. The strengths of this study are that it represents one of the largest published series of AAPL and reports on both clinical and quality of life outcomes for the patients involved. The relatively small number of patients and short duration of follow up were the main limitations. This study demonstrates a role for AAPL in the management of ESRD and a need for further randomized research into AAPL access techniques.

## Conclusion

We conclude that IAAPL represents a good alternative vascular access for haemodialysis in frail patients who have no conventional AV access options and for whom AV access and CVC lines are contraindicated. Good patency rates can be achieved and although complications including thrombosis and infection do occur, they do not commonly lead to limb compromise. IAAPL appears to be more effective and at least as safe as other tertiary vascular access techniques. More information from larger observational studies and randomised control trials directly comparing AAPL with other access methods would improve our understanding of exactly where this technique fits into the catalogue of vascular access techniques.

### What is known about this topic

- *It was known that arterio-arterial access is complex access with wide range of morbidity and considered inferior to other tertiary lines as the tunneled central lines*.

### What this study adds

- *Our results proved that IAAPL as one of the subtypes of arterio-arterial access is a good and safe alternative vascular access with minimal morbidity and mortality*.
